# Multiferroic Hysteresis Loop

**DOI:** 10.3390/ma10111318

**Published:** 2017-11-17

**Authors:** Alexander Ruff, Alois Loidl, Stephan Krohns

**Affiliations:** Experimental Physics V, Center for Electronic Correlations and Magnetism, University of Augsburg, 86159 Augsburg, Germany; alois.loidl@physik.uni-augsburg.de

**Keywords:** multiferroicity, LiCuVO_4_, spin-driven improper ferroelectricity, hysteresis in magnetic fields, multiferroic hysteresis Loop

## Abstract

Multiferroics, showing both ferroelectric and magnetic order, are promising candidates for future electronic devices. Especially, the fundamental understanding of ferroelectric switching is of key relevance for further improvements, which however is rarely reported in literature. On a prime example for a spin-driven multiferroic, LiCuVO_4_, we present an extensive study of the ferroelectric order and the switching behavior as functions of external electric and magnetic fields. From frequency-dependent polarization switching and using the Ishibashi-Orihara theory, we deduce the existence of ferroelectric domains and domain-walls. These have to be related to counterclockwise and clockwise spin-spirals leading to the formation of multiferroic domains. A novel measurement—multiferroic hysteresis loop—is established to analyze the electrical polarization simultaneously as a function of electrical and magnetic fields. This technique allows characterizing the complex coupling between ferroelectric and magnetic order in multiferroic LiCuVO_4_.

## 1. Introduction

In the last years, multiferroic materials established a very important field of materials science as they host inherent functionalities for novel electronic and magnetic devices [1]. Among these materials, those who exhibit both ferroelectric and (anti-)ferromagnetic order, are most prominent as they usually exhibit large magnetoelectric effects [2,3]. Controlling the magnetic order via an electric field and vice versa is a challenging task. Especially, systems with spin-driven ferroelectric order formed by spiral or helical spin structures enable this approach [4,5,6]. The electrical polarization arises directly from the non-collinear spin structure, for which LiCuVO_4_ is a prototypical example [4,6,7,8]. As proposed, e.g., in Refs. [9,10,11], the presence of tilted spins (*S_i_* and *S_i+_*_1_) at neighboring atomic sites (*i* and *i* + 1) breaks the inversion symmetry via spin-orbit coupling and is the microscopic mechanism for multiferroicity in these systems. This spin-driven improper ferroelectricity leads to the following relation for the electrical polarizat ion: *P* ∝ *e* × *Q*, where *Q* denotes the propagation vector of the spin spiral and *e* = (*S_i_* × *S_i+_*_1_) corresponds to the spiral axis, i.e., the normal vector of the spiral spin plane [7,10,11,12].

The spin-driven multiferroic compound LiCuVO_4_ exhibits a complex (*H*,*T*)-phase diagram for the polarization at low temperatures [7]. An external magnetic field in *c* direction (*H*_1_ ≈ 2.5 T < *H* < *H*_2_ ≈ 7.5 T) is able to gradually induce conical spin structures continuously decreasing the spin-driven polarization along the *a* direction, perpendicular to the external field. For increased magnetic fields (*H* > *H*_2_), a modulated collinear spin structure is established [13], which suppresses the helical spin state and electric polarization. Without an external magnetic field, the spin spiral in LiCuVO_4_ is formed below *T_N_* = 2.5 K in the *ab*-plane (spiral axis *e* ‖ *c*) and propagates in the crystallographic *b* direction (i.e., *Q* ‖ *b*) [7,8]. As predicted by theory (e.g., Refs. [10,12]) and confirmed by experiments (e.g., Refs. [7,14]) this leads to a ferroelectric polarization along *P* ‖ *a*. Above *H*_1_, *e* aligns along the external magnetic field direction, which is accompanied by spin flops if *H* is not along the easy axis (i.e., *c*). This allows switching the direction of the electrical polarization according to *P* ∝ *e* × *Q*. Accompanied by the transition into the modulated collinear spin state, above *H*_2_ the ferroelectric state vanishes. Interestingly, not only the magnetic field has an impact on the polarization of a LiCuVO_4_ single crystal but also an external electrical field [14]. This field can switch the ferroelectric polarization in *a* direction from +*P_a_* to −*P_a_* implying that the spin helicity switches from clockwise to counter-clockwise and vice versa. Such ferroelectric hysteresis loops have only rarely been documented in spin-spiral multiferroics [3,4,14,15,16].

In the present work, we thoroughly analyze the electric and magnetic field dependent ferroelectric hysteresis loops of single crystalline LiCuVO_4_. Special emphasis is put on two aspects: firstly, the frequency dependence of ferroelectric hysteresis shows that the polarization varies with respect to frequency and coercive field. We provide a fundamental basis, using the Ishibashi-Orihara theory for domain-wall movements [17], to explain the presence of multiferroic domains (clockwise and counterclockwise spin-spirals). This allows further insights into the dynamics of multiferroic switching processes. Secondly, on LiCuVO_4_ we demonstrate a novel multiferroic hysteresis loop measurement, which enables unraveling the complex coupling of ferroelectric and magnetic order, e.g., in the vicinity of the critical magnetic field *H*_2_. So far, only magnetic biasing fields were used for ferroelectric hysteresis loop measurements in multiferroics [14,18].

## 2. Results and Discussion

**Figure 1:**
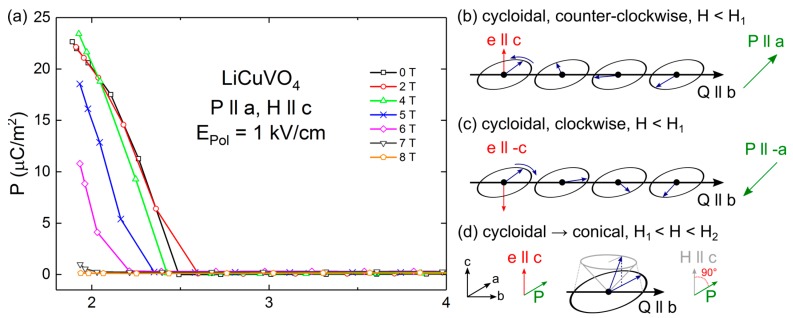
(**a**) Temperature dependent polarization along the *a* direction of a LiCuVO_4_ single crystal, measured during heating after a poling field of 1 kV/cm was applied while cooling. The pyrocurrent measurements were performed for various magnetic fields (up to 8 T) along the *c* direction; (**b**–**d**) illustrate possible spin-spiral configurations in LiCuVO_4_, also including the directions of polarization, applied magnetic field, spiral-axis and the modulation direction of the spin-spiral.

Figure 1a shows the temperature dependent polarization along the *a* direction, measured after polarizing the sample during cooling down to 1.8 K with an electric field of 1 kV/cm. In addition, the polarization was determined for different magnetic fields up to 8 T applied in *c* direction of the sample. A polarization of up to 24 µC/m^2^ for *H* < 4 T at low temperatures, for this specific measurement configuration is explained in terms of ferroelectric ordering, as described in Refs. [7,14]. For instance, in the absence of an external magnetic field, the polarization appears at the long-range magnetic order at *T_N_* = 2.5 K. For magnetic fields exceeding 2 T, the ferroelectric transition shifts to lower temperatures, which is in perfect agreement with reports of anomalies in temperature dependent dielectric constants [14]. Finally, the electrical polarization vanishes for magnetic fields above *H*_2_ coinciding with the paraelectric phase as a consequence of the modulated collinear spin state [7]. Hence, single crystalline LiCuVO_4_ investigated in this work is an illuminating example for spin-driven ferroelectric ordering for *T* < *T_N_*. The Figure 1b–d illustrates possible spin-spiral states of LiCuVO_4_ for zero and applied magnetic fields. We assume that for *H* < *H*_1_ (Figure 1b–c) purely cycloidal spin states in the *ab*-plane exist, which allow the switching of the electrical polarization from *+P* to *−P* in the *a* direction due to the helicity of the spin-spiral (rotation sense). These are the so-called clockwise and counter-clockwise spin helicities. In applied magnetic fields *H*_1_ < *H* < *H*_2_ along the c direction, the spin-spiral gradually transforms into a transverse conical configuration (Figure 1d). In the framework of the spin current model [10], the polarization in *a* direction arises due to a fictitious electric field, which is formed via spin current contributions of to the canted spin states projected onto the *ab*-plane [6]. The polarization derived from magnetocurrent measurement (i.e., measuring the pyrocurrent signal at constant temperature but changing magnetic field) [7] confirms this assumption, as the polarization changes from 0 to 24 µC/m^2^ between *H*_2_ and *H*_1_.

An inherent property of conventional ferroelectricity by definition is the switchability of the spontaneous electric polarization by an external electric field. Ruff et al. [14] demonstrated that even for LiCuVO_4_ the improper ferroelectric order could be controlled by an electric field. As a consequence of the relation *P* ∝ *e* × *Q*, the spin helicity (containing both modulation direction and spin spiral axis) of multiferroic LiCuVO_4_ has to switch between counterclockwise and clockwise direction [5,16]. Here, we investigate the electric polarization of these multiferroic domains as a function of the frequency of the applied electric switching pulse. The switching kinetics in conventional ferroelectrics are often interpreted using the Kolmogorov-Avrami-Ishibashi (KAI) model [19,20,21,22]. In this case, ferroelectric domains grow unrestrictedly from nucleation centers in an applied electric field. While switching the polarization, the domains start to overlap. Hence, the overall switched volume fraction is based on switching time, density of nuclei of reversed domains, mobility of domain walls, dimension of domain growth, and the impact of the electric field on moving domains. From the KAI model, Ishibashi and Orihara (IO) [17,20] derived a more simplified scenario, especially in the case of deterministic nucleation. Here, the volume fraction of reversed polarization depends purely on the frequency of the applied field and its waveform (normally sinusoidal). It turns out that the analysis of coercive fields derived from hysteresis-loops measurements performed with various frequencies can provide strong hints for the underlying ferroelectric switching mechanism [20].

**Figure 2:**
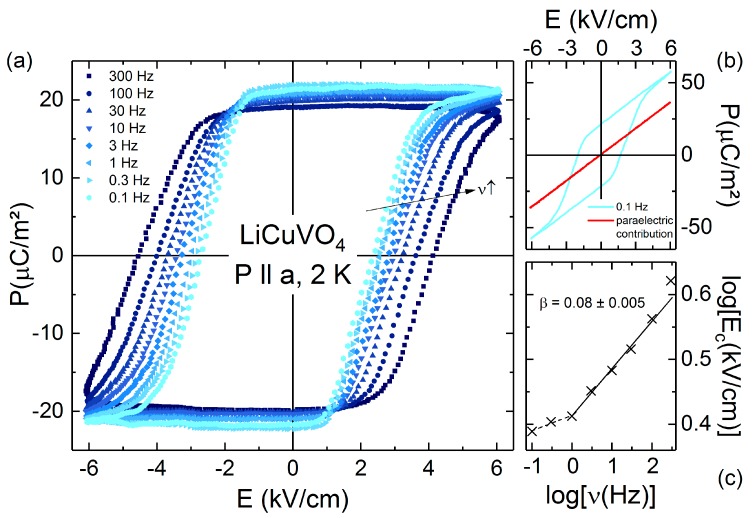
(**a**) Frequency dependent ferroelectric hysteresis loops of a LiCuVO_4_ single crystal, with the dielectric background subtracted; (**b**) Raw data of hysteresis loop for 0.1 Hz and respective *paraelectric* contribution (red line). For (**a**,**b**) The polarization *P* was measured along the *a* direction at *T* = 2 K and in electric fields *E* up to 6 kV·cm^−1^; (**c**) The inset shows a double-logarithmic representation of the coercive field *E_c_* vs. frequency *ν*. The line denotes a fit derived from the IO model resulting in a slope of *β* = 0.08 (see text).

In the scope of the IO scenario, we conduct a thorough analysis of frequency dependent (0.1 Hz < *ν* < 300 Hz) ferroelectric hysteresis loops *P*(*E*) of multiferroic LiCuVO_4_ as shown in Figure 2a for *T* = 2 K (no magnetic field applied). For the lowest frequency (*ν* = 0.1 Hz) of the applied sinusoidal electric field pulse, a fully saturated hysteresis loop emerges. Figure 2b shows the raw data of a hysteresis loop measurement. A tilt of the hysteresis loops arise from linear capacitance contributions, which is subtracted for all hysteresis loops within this manuscript. For LiCuVO_4_ positive-up-negative-down measurement reported in Ref. [14] exclude extrinsic effects, e.g., leakage current, giving rise to an artificial hysteresis loop [23,24]. The remnant polarization of about 22 µC/m^2^ confirms the polarization derived from pyrocurrent measurements (Figure 1a). With increasing frequency, the remnant polarization only slightly decreases, while the coercive field rises from *E_c_* (0.1 Hz) = 2.6 kV/cm to *E_c_* (300 Hz) = 4.3 kV/cm. Figure 2c presents this ν-dependence of *E*_c_ in a double-logarithmic scale. For higher frequencies *ν* > 1 Hz, log [*E_c_*(*ν*)] shows an almost linear increase in the log (*ν*) representation. For *ν* = 300 Hz, slight deviations are expected as the full saturation is not reached when applying an electric field pulse of *E_max_*(300 Hz) = 6 kV/cm (c.f. Figure 2a). In the scope of the IO model, *E*_c_ should follow a simplified power law relation: *E*_c_ ∝ *ν^β^* [20]. We use this model to describe *E_c_*(*ν*) and determine a *β*-parameter of 0.08 (±0.005), which is quite similar to *β*-values of domain-wall motion in conventional ferroelectrics, like PZT (*β* = 0.05) [25] and SBT (*β* = 0.12) [26]. Hence, the frequency dependent hysteresis loops of LiCuVO_4_ can be comprehensibly explained in the framework of the IO model. Consequently, the volume fraction of reversed polarization has to be directly linked to the magnetic order of counterclockwise and clockwise spin helicity. Thus, multiferroic domains are formed in LiCuVO_4_, which can be controlled by an external electric field.

**Figure 3:**
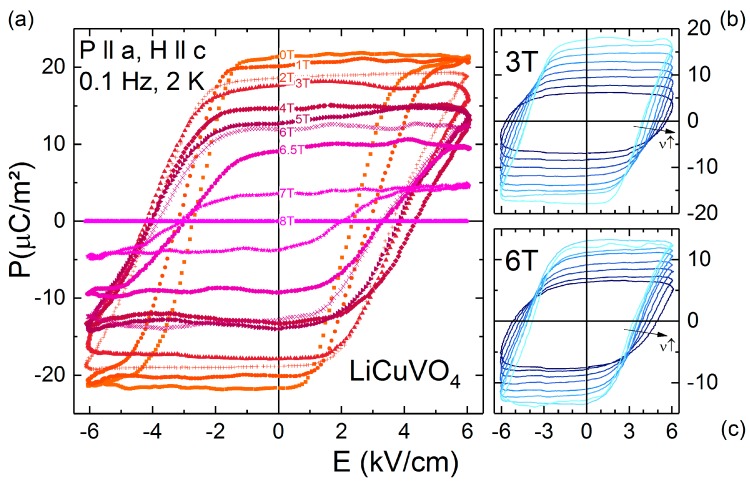
(**a**) Ferroelectric hysteresis loops of a LiCuVO_4_ single crystal measured at 2 K and 0.1 Hz in electrical fields up to 6 kV·cm^−1^ (with background subtracted); (**b**,**c**) show frequency dependent hysteresis loops (0.1 Hz < *ν* < 300 Hz) in magnetic fields of 3 T and 6 T, respectively. For (**a**–**c**), *P* is measured along the *a* direction. Static magnetic fields up to 8 T are applied along the *c* direction.

However, not only electric fields have an impact on the multiferroic domains but also applied magnetic fields do so. Figure 3a–c shows *P*(*E*) measured in static external magnetic fields up to 8 T. The magnetic field *H* is applied in the direction of the spiral axis *e* ‖ *c*, allowing *P* ‖ *a* even for *H*_1_ < *H* < *H*_2_ [14]. For the frequency of *P*(*E*) in Figure 3a we chose *ν* = 0.1 Hz, because the magnetic field enhances *E_c_*. So, if *E*_c_ is rather low, a fully saturated hysteresis loop can be achieved even in the presence of applied magnetic fields. For increasing magnetic fields, but still below *H*_1_, the coercive field strongly increases (from 2.5 kV/cm at 0 T to 3.9 kV/cm at 3 T) and the slope of the loop at *E_c_* flattens slightly. So, no fully saturated hysteresis loop can be achieved by the applied electric fields for 2 T < *H* < 7 T and the remnant polarization *P*_r_ decreases. In the regime *H*_1_ < *H* < *H*_2_ the remnant polarization declines towards zero for *H* > *H*_2_. In contrast, the coercive field has a reversal point at about 4 T leading to a decreasing *E_c_* for higher magnetic fields. The linear behavior of *P*(*E*) at *H* = 8 T points towards the absence of non-linear contributions, which denotes the capacitive background of the complete system (sample and measurements devices). Hence, this curve was used as *background* (i.e., paraelectric contribution) for all other measurements to determine the intersection revealing the coercive fields. So, in a nutshell, we observe that external magnetic fields lead to a strong decrease of the remnant polarization of multiferroic LiCuVO_4_. It seems that the coupling of the external magnetic field on the spin spiral impedes, especially if the magnetic field exceeds *H*_1_, the switchability (i.e., increasing *E_c_*) of the multiferroic domains, which are accompanied with multiferroic domain-wall movements. Indeed, frequency dependent *P*(*E*) loops with applied static magnetic fields *H* ‖ *c* of 3 T and 6 T (Figure 3b–c), at increasing external magnetic fields and higher frequencies of the *P*(*E*) loops reveal a strongly reduced *P_r_* and a shift of *E_c_* to higher values. However, probably the applied electrical field is too low to reach saturation polarization. It seems plausible that, due to the multiferroicity, the external magnetic field influences the domain-wall motion leading to deviations of the simple IO model.

**Figure 4:**
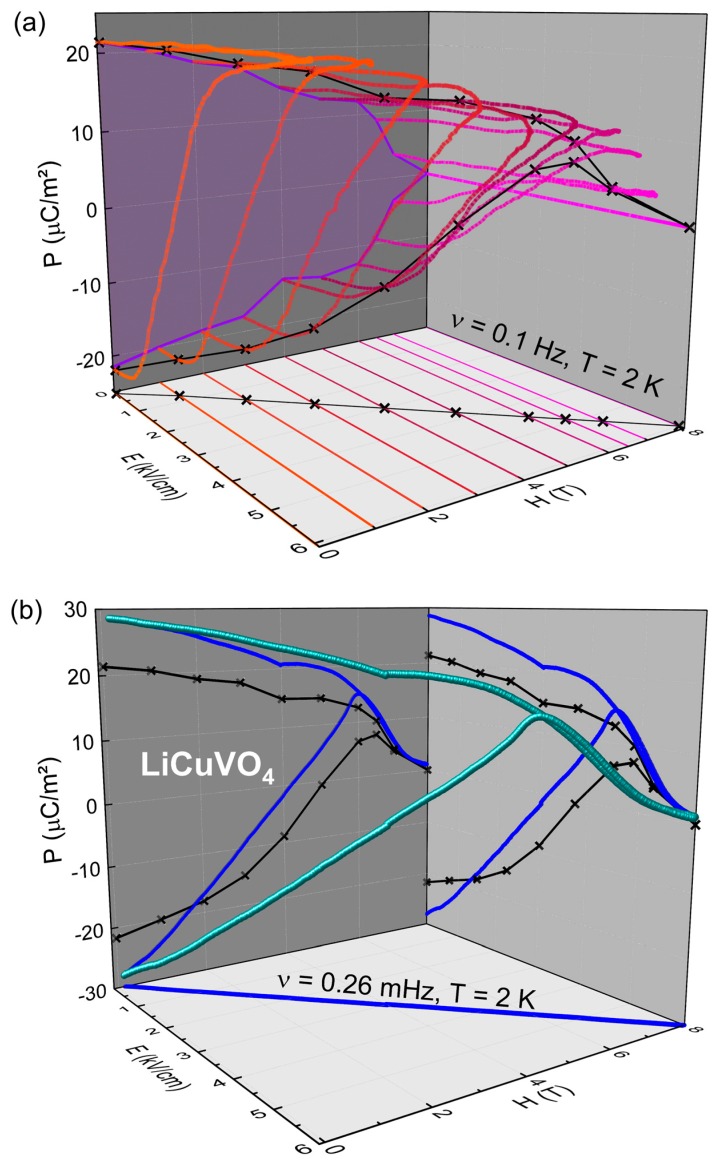
(**a**) *P*(*E*) loops of a LiCuVO_4_ single crystal, which are the same as shown in Figure 3. The color-coded lines (orange to magenta) represent the positive half of the hysteresis loops at static magnetic field. The crosses depict the intersection with the bisecting line in the *E*,*H*-plane. The *P*-values of these intersections forms a multiferroic hysteresis loop derived in static magnetic fields (shown also in (**b**)); (**b**) Multiferroic hysteresis loop of a LiCuVO_4_ single crystal measured at *T* = 2 K and 0.26 mHz, again with *P* along the *a* direction and magnetic fields along the *c* direction. The dark-blue lines are projections of the multiferroic hysteresis loop in *P*(*E*), *P*(*H*) and within *H*,*E* planes.

Hence, we performed a novel experiment by measuring the polarization when varying simultaneously magnetic and electric fields with the same rise-time and waveform. This multiferroic hysteresis loop is compared to *P*(*E*) loops detected in static external magnetic fields. Figure 4a shows the results of hysteresis-loop measurements of Figure 3a in a *P*(*E*,*H*) representation (for clarity, only half of the loops are shown as the polarization behavior is almost symmetric; c.f., Figure 3a). The lines in the *E*,*H*-plane denote the applied static magnetic field. As proof of concept, we show in Figure 4b a multiferroic hysteresis loop (MHL) at low frequencies, to reach saturation polarization, and in magnetic fields up to 8 T to detect the ferroelectric to paraelectric transition at *H*_2_. We apply an electrical pre-poling pules at zero magnetic field to start at *−P_r_*. A line spanned between the origin and *P*(6 kV cm^−1^, 8 T) is used to subtract the paraelectric contribution. The same limits (*E_max_* = 6 kV cm^−1^ and *H_max_* = 8 T) are used to derive a multiferroic hysteresis loop from intersections of *P*(*E*) loops (static magnetic fields) with a theoretical bisecting line in the *E*,*H*-plane (c.f. black line and crosses in Figure 4a). The derived *P*-values of that loop reflect roughly the evolution of the dynamic multiferroic hysteresis loop (Figure 4b). The switching process of the polarization from *−P_r_* to *+P_r_* takes place over a broad range of electrical and magnetic fields differing significantly from typical *P*(*E*) loops in static magnetic fields below *H*_2_. Approaching *H*_2_ the MHL shows a clear peak like feature (i.e., *E* = 4.6 kV and *H* = 6.1 T), indicating an accelerated rise of *P* before the polarization approaches zero in the non-collinear state. Above *H*_2_, the polarization resembles the pure paraelectric background (about 36 µC/m^2^ at 2 K and for 8 T in the raw data). Interestingly, for decreasing (*E*,*H*)-fields, the peak-feature in *P* rises again. Below *H*_2_, the overall *P* for zero electrical and magnetic fields reaches a value of about 29 µC/m^2^, which agrees with results of pyrocurrent (Figure 1) and magnetocurrent (Figure 4a of Ref. [7]) measurements. A pronounced MHL requires the possibility to switch the polarization. For the cycloidal state, this is allowed due to the formation of clockwise and counter-clockwise spin spirals. However, for *H* > *H*_1_ we assume the emergence of a transverse conical spin structure. The projection of the canted spins *S_i_* and *S_i+_*_1_ onto the *ab* plane gradually reduces the absolute value of *P_r_* in *a* direction. A polarization reversal is still possible as the conical spin spiral can rotate again clockwise or counter-clockwise.

The MHL technique allows analyzing the “dynamic” polarization reversal process as function of electric and magnetic fields. In Figure 4a, the violet-shaded area in the *P*(*H*)-plane denotes the switchable polarization for an applied electric field of *E_max_* = 6.1 kV cm^−1^ and *ν* = 0.1 Hz in static magnetic fields. The MHL-curve for *ν* = 0.26 mHz in Figure 4b shows the polarization reversal of multiferroic domains starting at *−P_r_*. Even small changes in the applied electric and magnetic fields reduce the overall polarization. One possible explanation is that, for these low magnetic fields, the transverse conical spin state starts to develop, despite the fact that this field is still below the magnetic anisotropy field, which is in the order of 2.5 T. The accompanied coercive field as a function of magnetic and electric field is of order 3.6 kV cm^−1^ at 4.1 T. This is followed by the aforementioned peak feature in *P*(*E*,*H*) approaching *H*_2_. Interestingly, forward and reverse poling result in almost the same polarization, pointing to a saturated state of clockwise spins spirals. Taking into account the significant decrease of *E_c_* for the 7 T curve in Figure 4a, it seems possible that, in the transverse conical state close to the collinear spin configuration at *H*_2_, the spin helicity can be switched by lower electric fields. This helicity then remains even if both electric and magnetic fields are turned off. One can speculate that this complex interplay of electric and magnetic order in multiferroic LiCuVO_4_ allows poling of *P* by smaller electric fields, if the magnetic field close to the collinear spin state is applied. Further detailed measurements are required to analyze especially the frequency dependent dynamics of this switching process. Finally, a distinct benefit of the novel MHL technique is the precise measurement within a certain *E*,*H* parameter set enabling the analysis of multiferroic coupling in the vicinity of critical electric and magnetic fields.

## 3. Summary

In summary, we have performed a thorough characterization of the switching polarization of LiCuVO_4_, which is a prime-example of spin-driven multiferroicity, by investigating the ferroelectric hysteresis loops as function of frequencies and magnetic fields below the ordering temperature. From the frequency dependence of the coercive fields and using the Ishibashi-Orihara model, we conclude the existence of ferroelectric domains. Magnetic domains of clockwise and counter-clockwise spin-spirals representing rarely observed multiferroic domains accompany these ferroelectric domains. To determine the complex interplay of this multiferroic state, we establish a novel technique: multiferroic hysteresis loop measurement. Therefore, both fields vary with the same waveform and frequency allowing the analysis of *P*(*E*,*H*)-loops. From these measurements, we deduce the existence of switchable polarization in the cycloidal (*H* < *H*_1_) and in the transverse conical state (*H*_1_ < *H* < *H*_2_). In the latter case, the overall polarization in *a* direction is gradually reduced, due to two mechanisms: first, the external magnetic fields hamper the switching of the spin helicity, leading to a strong increase in the electrical coercive fields; and, secondly, the formation of a conical spin arrangement reduces the absolute value of the polarization. However, in the vicinity of *H*_2_ the coercive field is strongly reduced, which allows switching the helicity by even smaller electric fields. Hence, this novel technique enables unraveling complex coupling phenomena in multiferroic systems, and, if a magnetic field of high rise-time is available, also frequency dependent multiferroic hysteresis loop measurements, which can provide promising insights to the switching kinetics of multiferroic domains.

## 4. Materials and Methods

### 4.1. Sample Preparation

Single crystals of the orthorhombic distorted spinel compound LiCuVO_4_ were grown from a LiVO_3_-based flux, as described in detail in Refs. [27,28]. Even a slightly variation in the composition results in different sample properties. Therefore, the single-phase and stoichiometry of the crystal were checked by X-ray diffraction and differential dissolution technique, respectively. A crystal with almost ideal Li and Cu sublattices [13] and a size of approximately 3 × 1 × 1 mm^3^ was chosen and oriented by Laue diffraction technique.

### 4.2. Polarization Measurements

The pyroelectric current and the hysteresis-loop measurements were performed for electrical fields along the *a* direction and are in perfect agreement with previous measurements on that sample [7,14]. For polarization measurements, silver paint contacts were applied to the single crystal in sandwich geometry to measure *P* along the *a* direction. For measurements between 1.5 and 30 K and in external magnetic fields up to 9 T, a Physical Property Measurement System (Quantum Design, San Diego, CA, USA) and a cryostat equipped with a superconducting magnet (Oxford Instruments, Oxon, UK) was used. To probe the ferroelectric order, the pyroelectric current at fixed magnetic fields was measured as a function of temperature between 1.8 K and 30 K, utilizing a Keithley Electrometer 6517 A (Keithley, Cleveland, OH, USA). A typical temperature rate of 5 K/min was used. The spontaneous polarization was obtained by integrating the current over the time. Further, in order to align the ferroelectric domains, a poling field of about 1 kV/cm was applied during cooling of the sample through the magnetic transition temperature. Hysteresis-loop measurements were made using an Aixacct TF2000 ferroelectric analyzer (aixACCT Systems GmbH, Aachen, Germany) equipped with a high-voltage booster. All hysteresis curves show a certain slope, which is independent of temperature and external magnetic fields, at least in the measured ranges of the present work. This is due to the contribution of a linear capacity, which can be neglected by subtraction of a straight line with an appropriate slope leaving only the non-linear electric contributions. In this manuscript, we use the corrected curves to evaluate the frequency dependent coercive fields *E*_c_. Furthermore, the coercive fields were calculated using *E*_c_ = (|*E*_c+_| + |*E*_c−_|)/2, where *E*_c+_ and *E*_c−_ are the positive and negative coercive fields.

### 4.3. Multiferroic Hysteresis Loop

For the multiferroic hysteresis loop measurement *P(E*,*H)*, simultaneously an electric and magnetic field with the same frequency was applied to the sample while the polarization was determined. The frequency was limited by the maximum sweeping rate of the magnetic field of 0.5 T/min of the Oxford Cryostat.

## References

[1] Fiebig M., Lottermoser T., Meier D., Trassin M. (2016). The evolution of multiferroics. Nat. Rev. Mater..

[2] Fiebig M. (2005). Revival of the magnetoelectric effect. J. Phys. D Appl. Phys..

[3] Hill N.A. (2000). Why Are There so Few Magnetic Ferroelectrics?. J. Phys. Chem. B.

[4] Cabrera I., Kenzelmann M., Lawes G., Chen Y., Erwin R., Gentile T.R., Leao J.B., Lynn J.W., Rogado N., Cava R.J. (2009). Coupled Magnetic and Ferroelectric Domains in Multiferroic Ni_3_V_2_O_8_. Phys. Rev. Lett..

[5] Yamasaki Y., Sagayama H., Goto T., Matsuura M., Hirota K., Arima T., Tokura Y. (2007). Electric Control of Spin Helicity in a Magnetic Ferroelectric. Phys. Rev. Lett..

[6] Tokura Y., Seki S. (2010). Multiferroics with Spiral Spin Orders. Adv. Mater..

[7] Schrettle F., Krohns S., Lunkenheimer P., Hemberger J., Büttgen N., Krug von Nidda H.-A., Prokofiev A.V., Loidl A. (2008). Switching the ferroelectric polarization in the S=1/2 chain cuprate LiCuVO_4_ by external magnetic fields. Phys. Rev. B.

[8] Naito Y., Sato K., Yasui Y., Kobayashi Y., Kobayashi Y., Sato M. (2007). Ferroelectric Transition Induced by the Incommensurate Magnetic Ordering in LiCuVO_4_. J. Phys. Soc. Jpn..

[9] Gibson B.J., Kremer R.K., Prokofiev A.V., Assmus W., McIntyre G.J. (2004). Incommensurate antiferromagnetic order in the S = 1/2 quantum chain compound LiCuVO_4_. Physica B.

[10] Katsura H., Nagaosa N., Balatsky A.V. (2005). Spin Current and Magnetoelectric Effect in Noncollinear Magnets. Phys. Rev. Lett..

[11] Sergienko I.A., Dagotto E. (2006). Role of the Dzyaloshinskii-Moriya interaction in multiferroic perovskites. Phys. Rev. B.

[12] Mostovoy M. (2006). Ferroelectricity in Spiral Magnets. Phys. Rev. Lett..

[13] Büttgen N., Krug von Nidda H.-A., Svistov L.E., Prozorova L.A., Prokofiev A., Aßmus W. (2007). Spin-modulated quasi-one-dimensional antiferromagnet LiCuVO_4_. Phys. Rev. B.

[14] Ruff A., Krohns S., Lunkenheimer P., Prokovief A., Loidl A. (2014). Dielectric properties and electrical switching behaviour of the spin-driven multiferroic LiCuVO_4_. J. Phys. Condens. Matter.

[15] Kimura T., Lawes G., Goto T., Tokura Y., Ramirez A.P. (2005). Magnetoelectric phase diagrams of orthorhombic RMnO_3_ (R = Gd, Tb, and Dy). Phys. Rev. B.

[16] Tokura Y., Seki S., Nagaosa N. (2014). Multiferroics of spin origin. Rep. Prog. Phys..

[17] Ishibashi Y., Orihara H. (1995). A Theory of D-E Hysteresis Loop-Application of Avrami Model. Integr. Ferroelectr..

[18] Tokunaga Y., Taguchi Y., Arima T., Tokura Y. (2014). Magnetic Biasing of a Ferroelectric Hysteresis Loop in a Multiferroic Orthoferrite. Phys. Rev. Lett..

[19] Orihara H., Hashimoto S., Ishibashi Y. (1994). A Theory of D-E Hysteresis Loop Based on the Avrami Model. J. Phys. Soc. Jpn..

[20] Hashimoto S., Orihara H., Ishibashi Y. (1994). Study on D-E Hysteresis Loop of TGS Based on the Avrami-Type Model. J. Phys. Soc. Jpn..

[21] Kolmogorov A.N. (1937). A statistical theory for the recrystallization of metals. Izv. Akad. Nauk SSSR Ser. Mat..

[22] Avrami M. (1940). Kinetics of phase change. II transformation-time relations for random distribution of nuclei. J. Chem. Phys..

[23] Scott J.F. (2008). Ferroelectrics go bananas. J. Phys. Condens. Matter.

[24] Loidl A., Krohns S., Hemberger J., Lunkenheimer P. (2008). Bananas go paraelectric. J. Phys. Condens. Matter.

[25] Scott J.F., Ross F.M., Paz de Araujo C.A., Scott M.C., Huffman M. (1996). Structure and Device Characteristics of SrBi_2_Ta_2_O_9_-Based Nonvolatile Random-Access Memories. MRS Bull..

[26] Scott J.F. (2000). Ferroelectric Memories.

[27] Prokofiev A.V., Wichert D., Assmus W. (2000). Crystal growth of the quasi-one dimensional spin-magnet LiCuVO_4_. J. Cryst. Growth.

[28] Prokofiev A.V., Vasilyev I.G., Assmus W. (2005). Crystal growth of LiCuVO_4_: Influence of the flux composition and the growth temperature on the stoichiometry and perfection of the crystals. J. Cryst. Growth.

